# Genetically related genotypes of cowpea present similar bacterial community in the rhizosphere

**DOI:** 10.1038/s41598-022-06860-x

**Published:** 2022-03-02

**Authors:** Tayná Mendes de Albuquerque, Lucas William Mendes, Sandra Mara Barbosa Rocha, Jadson Emanuel Lopes Antunes, Louise Melo de Souza Oliveira, Vania Maria Maciel Melo, Francisca Andrea Silva Oliveira, Arthur Prudêncio de Araujo Pereira, Veronica Brito da Silva, Regina Lucia Ferreira Gomes, Francisco de Alcantara Neto, Angela Celis de Almeida Lopes, Maurisrael de Moura Rocha, Ademir Sergio Ferreira Araujo

**Affiliations:** 1 Plant Genetic Resource Group, Agricultural Science Center, Federal University of Piauí, Teresina, PI Brazil; 2 Center for Nuclear Energy in Agriculture, University of Sao Paulo, Piracicaba, SP Brazil; 3 Soil Microbial Ecology Group, Agricultural Science Center, Federal University of Piauí, Teresina, PI Brazil; 4 Laboratório de Ecologia Microbiana E Biotecnologia, Federal University of Ceará, Fortaleza, CE Brazil; 5 Soil Science Department, Federal University of Ceará, Fortaleza, CE Brazil; 6 Embrapa Meio Norte, Teresina, PI Brazil

**Keywords:** Microbial ecology, Microbiome, Plant breeding

## Abstract

Plant breeding reduces the genetic diversity of plants and could influence the composition, structure, and diversity of the rhizosphere microbiome, selecting more homogeneous and specialized microbes. In this study, we used 16S rRNA sequencing to assess the bacterial community in the rhizosphere of different lines and modern cowpea cultivars, to investigate the effect of cowpea breeding on bacterial community assembly. Thus, two African lines (IT85F-2687 and IT82D-60) and two Brazilian cultivars (BRS-Guariba and BRS-Tumucumaque) of cowpea were assessed to verify if the generation advance and genetic breeding influence the bacterial community in the rhizosphere. No significant differences were found in the structure, richness, and diversity of bacterial community structure between the rhizosphere of the different cowpea genotypes, and only slight differences were found at the OTU level. The complexity of the co-occurrence network decreased from African lines to Brazilian cultivars. Regarding functional prediction, the core functions were significantly altered according to the genotypes. In general, African lines presented a more abundance of groups related to chemoheterotrophy, while the rhizosphere of the modern cultivars decreased functions related to cellulolysis. This study showed that the genetic breeding process affects the dynamics of the rhizosphere community, decreasing the complexity of interaction in one cultivar. As these cowpea genotypes are genetically related, it could suggest a new hypothesis of how genetic breeding of similar genotypes could influence the rhizosphere microbiome.

## Introduction

The interaction between plants and microorganisms in the rhizosphere regulates several biological processes important to plants. The rhizosphere is known as the narrow zone of soil, which is driven by root traits, being a specific zone where thousands of microbial species live in close association with plants^1^. Particularly, studies about plant-microorganism interactions in the rhizosphere have focused on bacterial communities that represent the most versatile and diverse groups of microbes found in soils^2^. Recently, Geisen et al.^2^ have estimated more than one million bacterial species in soils acting on some important biological processes, such as primary production and nutrient cycling.

In the rhizosphere, the bacterial community interacts with plant roots and stimulates plant development, inhibits pathogens, and solubilizes nutrients, so conferring positive effects on plants^3^. On the other hand, different traits of the rhizosphere presented by distinct plants species can shape the pattern of the bacterial community. Indeed, different plant species^4^, the stage of development^5^, characteristics of root exudation^6^, and different cultivars^7,8^, can select specific bacterial groups and contribute to change diversity and richness of species. For instance, Araujo et al.^5^ assessed the influence of the stage of development in maize and cowpea on bacterial community in the rhizosphere and found that the structure and diversity of bacterial community varied significantly according to different plant species and, to a minor extent, to their development stage.

The influence of the rhizosphere on the response of the bacterial community in several plants species and conditions is well known, but it remains unclear how plant breeding shapes the assembly of the bacterial community in the rhizosphere. Plant breeding is a process involving the changes in plant traits to obtain superior characteristics, such as biotic and abiotic stress tolerance and grain yield^9^. This process is usually done with the use of advancing generations and selection of lines with the obtention of modern cultivars^10^. Although being positive to humans being, plant breeding promotes a reduction in the genetic diversity of plants^11^ and it can, consequently, select more homogeneous and specialized microbes^12^. Therefore, plant breeding can potentially modify the composition, structure, and diversity of the bacterial community in the rhizosphere. Indeed, previous studies have reported that plant breeding and the advance of generation significantly impacted rhizosphere microbial communities and network assembly^7,13,14^, promoting a homogenization and specialization of the bacterial community in the rhizosphere as influenced by the decreased genetic diversity^12,15,16^.

As an important tropical plant species, cowpea (*Vigna unguiculata* L. Walp.) has been submitted to intensive plant breeding, mainly to figure out modern cultivars with higher tolerance to biotic and abiotic factors and yield^17^. However, little attention has been paid to the effect of cowpea breeding on the bacterial community in the rhizosphere. The knowledge of the behavior of the bacterial community in the rhizosphere of cowpea is important since it could positively influence plant performance. Here, we hypothesized that the process of cowpea breeding would influence plants traits and consequently drive the pattern of the bacterial community. Thus, we used 16S rRNA sequencing to assess the bacterial community in the rhizosphere of different lines and modern cowpea cultivars, to investigate the effect of cowpea breeding on bacterial community assembly.

## Results

### Bacterial community structure and diversity

**Figure 1:**
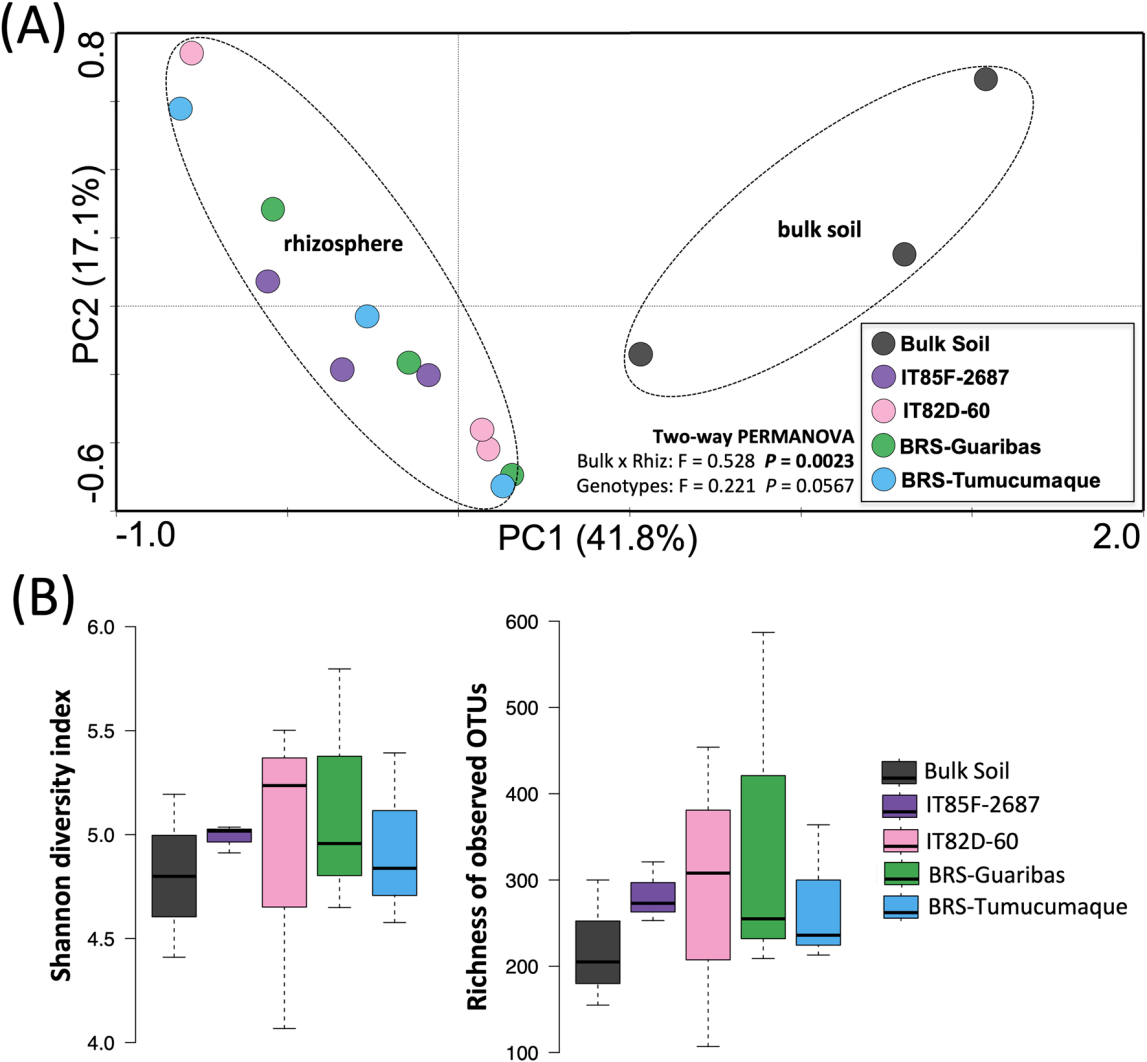
Structure and diversity of bacterial communities in bulk soil and rhizosphere of four cowpea genotypes based on the 16S rRNA gene. (**A**) Principal component analysis of the bacterial community structure. The dashed lines in the graph indicate significant clusters (PERMANOVA, *p* < 0.05). (**B**) Taxonomic diversity and richness based on OTU level at 97% of similarity. Error bars represent the standard deviation, and no differences were found between treatments, based on Tukey’s HSD test (*p* > 0.05).

The principal component analysis (PCA) explained 58.9% of the total variation in bacterial operational taxonomic units (OTU) of which 41.8% and 17.1% are displayed on the first two axes, respectively (Fig. 1A). As expected, the PCA showed significant differences in bacterial community structure between bulk soil and rhizosphere of cowpea genotypes (PERMANOVA Bulk x Rhizosphere F = 0.5281, *p* = 0.0023). However, no significant differences were found in bacterial community structure between the rhizosphere of the different cowpea genotypes (PERMANOVA Genotypes F = 0.221, *p* = 0.0567). The results also showed no differences in both richness and diversity of the bacterial community between each rhizosphere and the bulk soil (Fig. 1B). To compare the proportion of shared and exclusive OTUs between bulk soil and cowpea genotypes, Venn diagrams were used (Supplementary Figure S1). Bulk soil and rhizosphere (the pool of the four cultivars) shared only 16.6% of the OTUs (71 OTUs in total), while 15.2% (65) and 68.2% (292) were exclusive in bulk soil or rhizosphere, respectively. Regarding the differences between cowpea genotypes, 19% of the OTUs (69) were shared among all genotypes, while the cultivar BRS-Guariba and the line IT82D-60 showed the highest and lowest proportion of exclusive OTUs, respectively.

### Bacterial community composition

**Figure 2:**
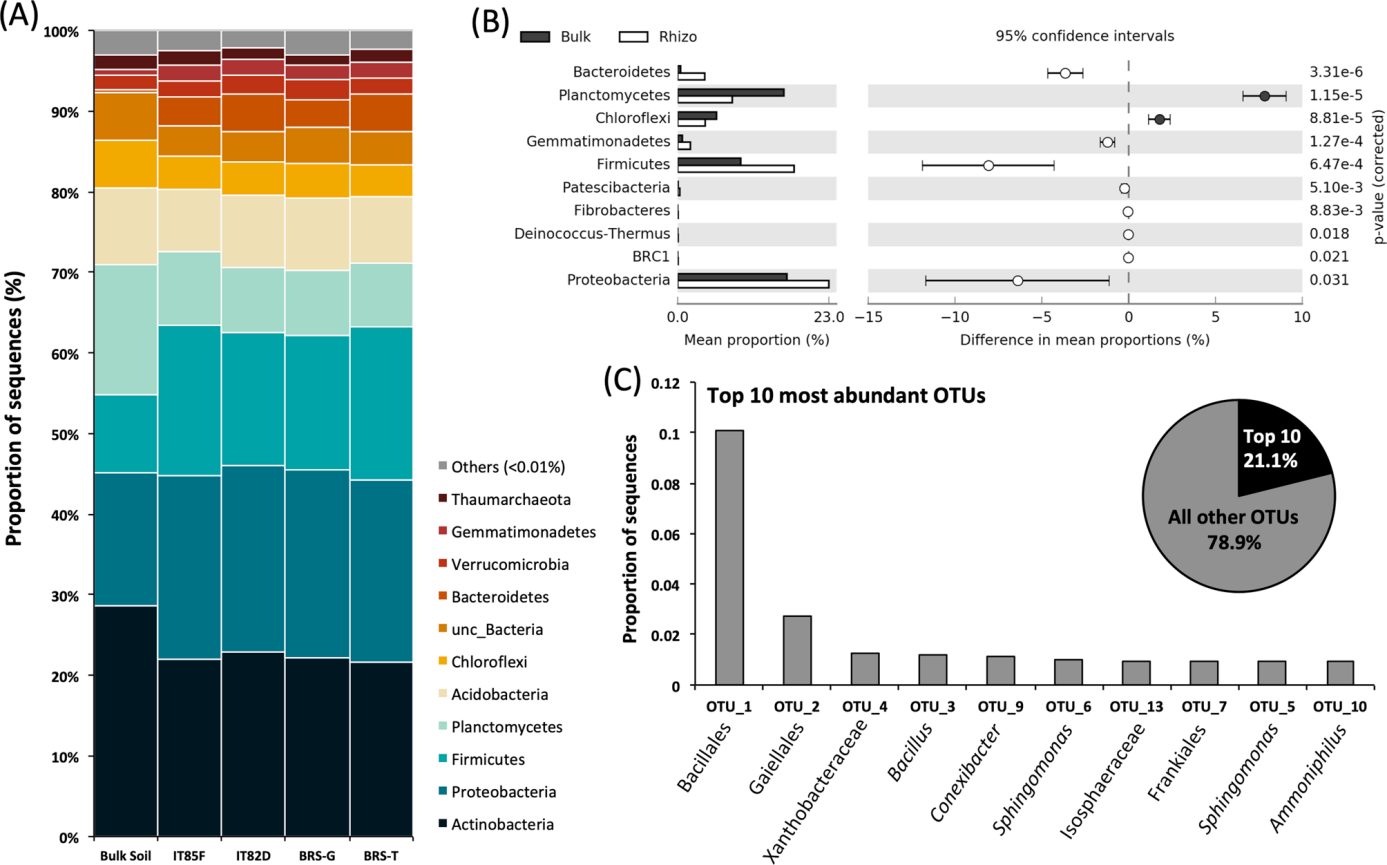
(**A**) General overview of the taxonomic composition of the bacterial community in bulk soil and rhizosphere of different cowpea genotypes. (**B**) Differential abundance of phyla between bulk soil and the cowpea rhizosphere based on the 16S rRNA gene. (**C**) Top ten most abundant OTUs found in all samples. The classification in the figure shows the lowest level of affiliation based on SILVA database at 97% of similarity.

The bacterial community was composed of 25 phyla, being dominated by Actinobacteria (23.5% of all the sequences), followed by Proteobacteria (23%), Firmicutes (16%), Planctomycetes (10%), Acidobacteria (8.5%), and Chloroflexi (4.5%) (Fig. 2A). However, the bacterial community composition presented different abundance according to bulk soil and rhizosphere, with enrichment of Proteobacteria, Firmicutes, Bacteroidetes, Gemmatimonadetes, among others (Fig. 2B). On the other hand, bulk soil presented a higher abundance of the phyla Planctomycetes and Chloroflexi. We also highlighted the top 10 most abundant OTUs, which together comprised about 21% of the total OTUs. The most abundant OTU was affiliated to the order Bacillales, followed by OTUs classified as Gaiellales, Xanthobacteraceae, *Bacillus*, *Conexibacter*, *Sphingomonas*, Isosphaeraceae, Frankiales, and *Ammoniphilus* (Fig. 2C).

**Figure 3:**
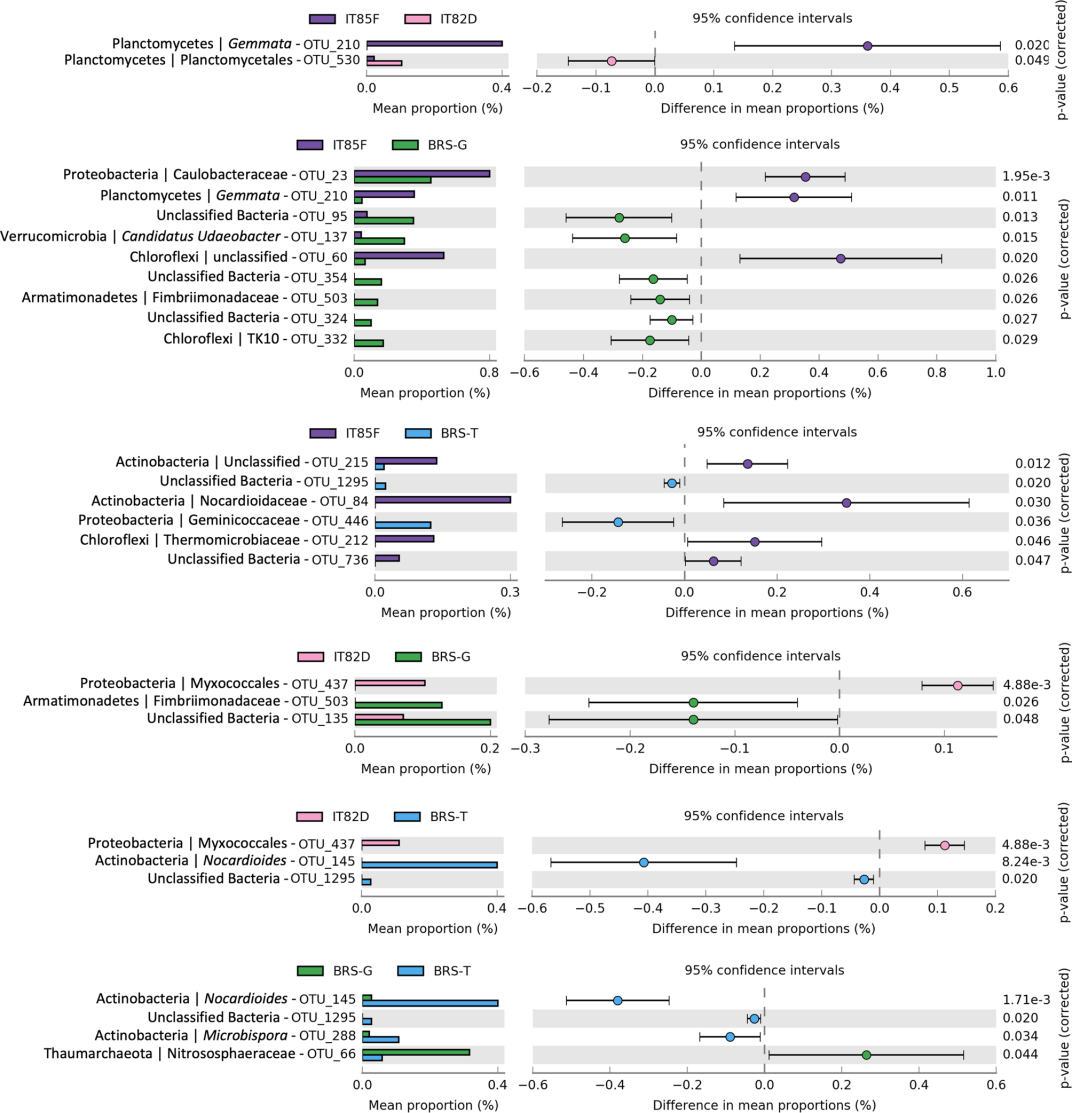
Scatter-plots showing the differential abundance of OTUs between the different cowpea genotypes. The significance is based on Welch's t-test with Benjamini–Hochberg correction (*p* < 0.05). The classification in the figure shows the lowest level of affiliation based on SILVA database at 97% of similarity.

We then conducted a pair-wise comparison between the rhizosphere of the different cowpea genotypes and the results showed an enrichment according to each genotype, suggesting a genotype-specific rhizospheric effect (Fig. 3). The line IT85F-2687 enriched Gemmata compared to line IT82D-60, which enriched Planctomycetes. Comparing the line IT85F-2687 and the cultivar BRS-Guariba, the first enriched Caulobacteraceae, Gemmata, and unclassified Chloroflexi, while the second one enriched unclassified bacterium, *Candidatus Udaeobacter*, Fimbriimonadaceae, and TK10. In the comparison between line IT85F-2687 and cultivar BRS-Tumucumaque, the first enriched unclassified Actinobacteria, Nocardioidaceae, Thermomicrobiaceae, and unclassified bacteria, while the second one enriched Geminicoccaceae. The line IT82D-60 enriched Myxococales as compared to cultivar BRS-Guariba, which enriched Fimbriimonadaceae and unclassified bacteria. When compared line IT82D-60 with cultivar BRS-Tumucumaque, the first enriched Myxococcales, while the second enriched *Nocardioides*. Finally, the cultivar BRS-Guariba enriched Nitrososphaeraceae, while the cultivar BRS-Tumucumaque enriched *Nocardioides* and *Microbispora*. In general, the differences between African lines and Brazilian cultivars were higher than within the African or Brazilian group.

### Correlation analysis

**Figure 4:**
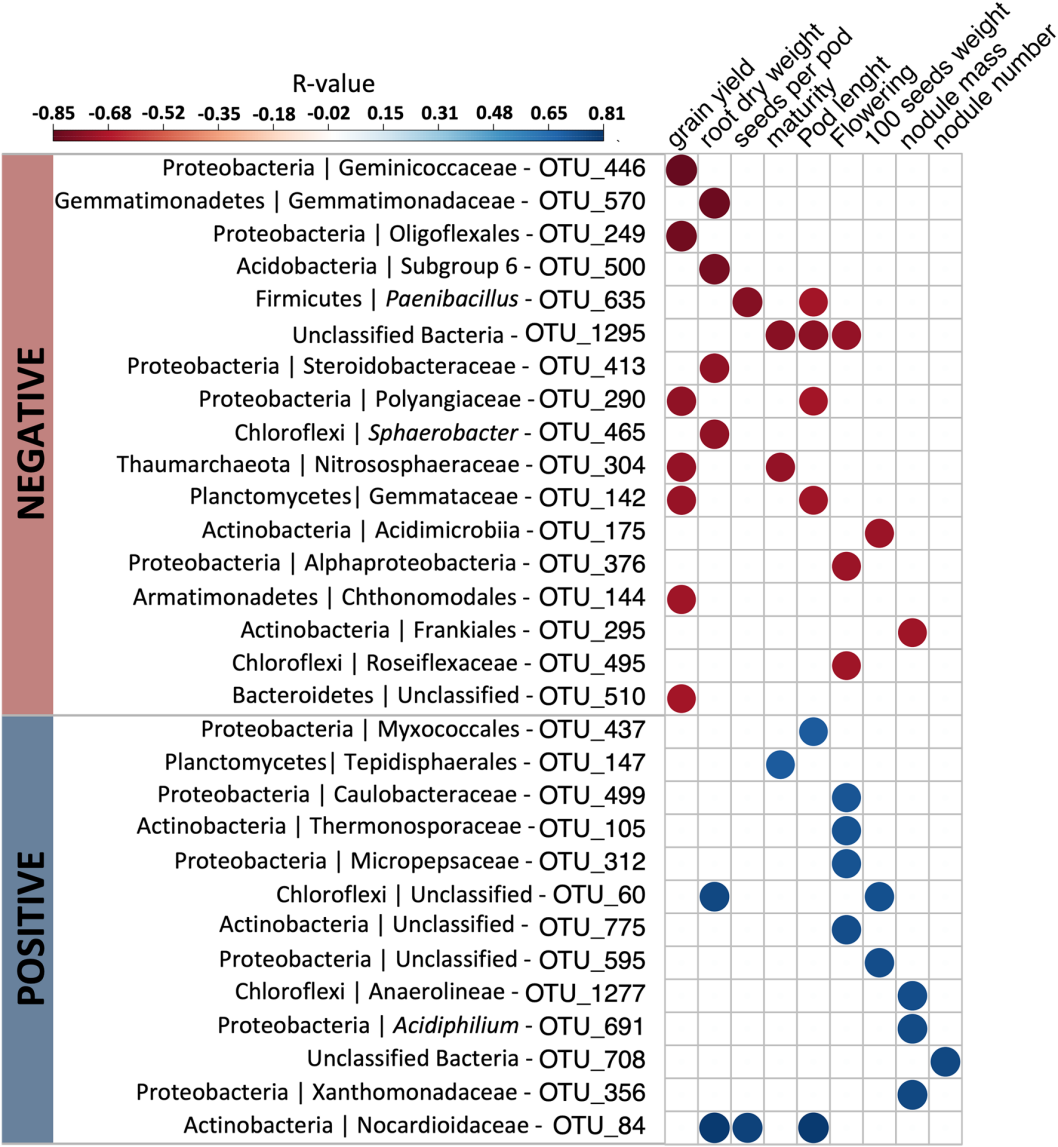
Heatmap showing the Spearman's rank correlation coefficients and statistical significance between OTUs and plant parameters. Blue and red colors indicate significant positive and negative correlations, respectively (*p* < 0.05).

To analyze the correlation between individual bacterial groups (at OTU level) and specific plant performance traits we calculated all possible Spearman’s rank correlations to identify possible bioindicators that influence cowpea plant growth and health (Fig. 4). The analysis showed that 17 and 13 bacterial OTUs presented negative and positive correlations with plant performance traits, respectively. More specifically, grain yield (7 negative correlations), root dry weight (4), and pod length (4) showed the highest numbers of negative correlation with bacterial groups. In contrast, flowering (4) and nodule mass (3) showed the highest number of positive correlations. When considering bacterial groups, Unclassified bacteria (3), *Paenibacillus* (2), Polyangiaceae (2), Nitrososphaeraceae (2), and Gemmataceae (2) presented negative correlation with cowpea genotypes traits, while Nocardioidaceae (3) and Unclassified Chloroflexi (2) showed positive correlations.

### Co-occurrence network

**Figure 5:**
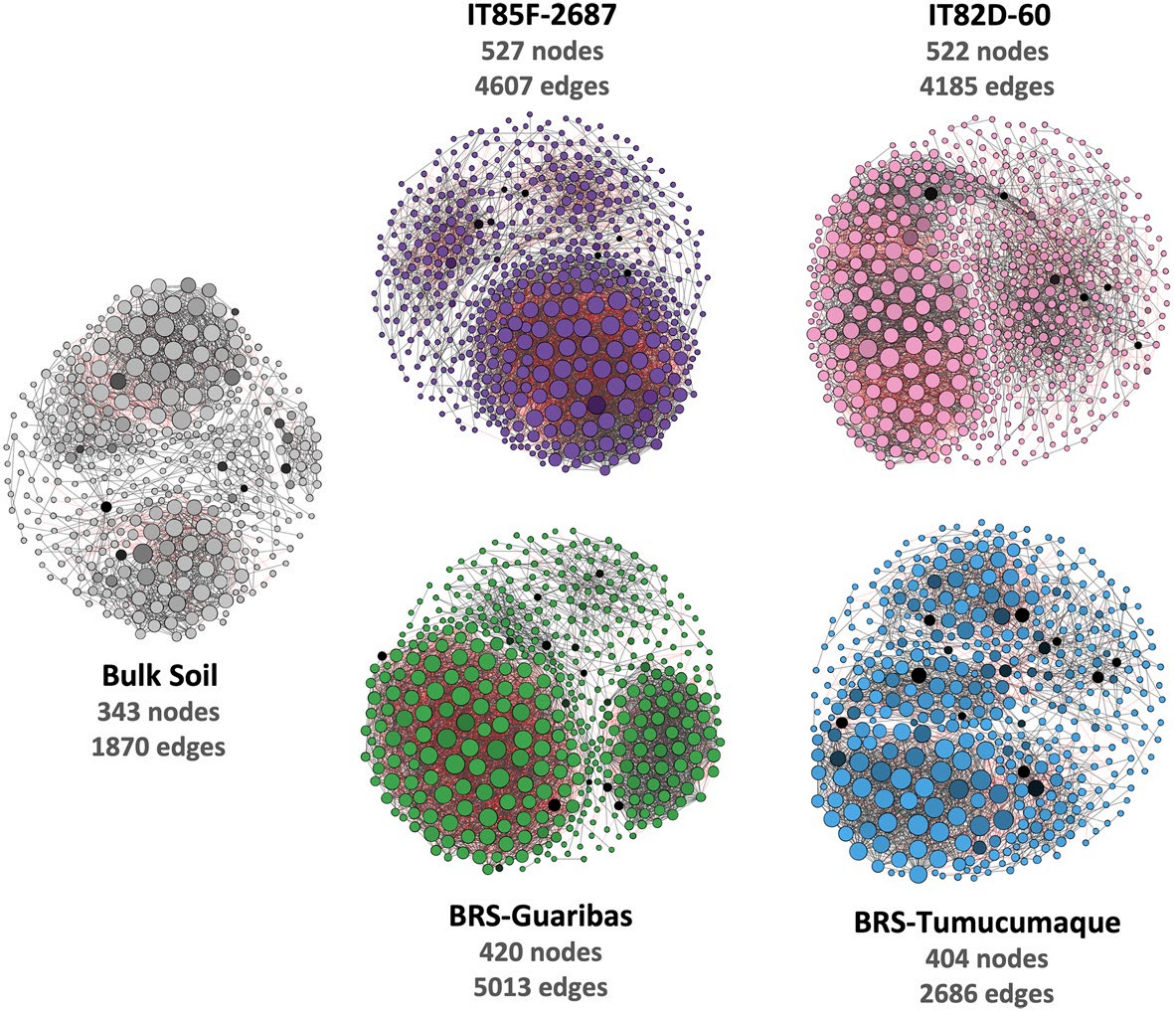
Network co-occurrence analysis of the bacterial communities in bulk soil and rhizosphere of four distinct cowpea genotypes based on the 16S rRNA gene. A connection stands for SparCC correlation with magnitude > 0.7 (positive correlation–black edges) or <  − 0.7 (negative correlation–red edges) and statistically significant (*p* ≤ 0.01). Each node represents taxa at OTU level, and the size of node is proportional to the number of connections (that is, degree). The color of the nodes is based on the betweenness centrality, where darker colors indicated higher values. The black nodes indicate the top five keystone species, depicted here as the nodes with the highest betweenness centrality.

The complexity of connections in the bacterial community was assessed by the co-occurrence network analysis (Fig. 5; Supplementary Table S1). Bulk soil and cowpea genotypes presented distinct network compositional and topological features. As expected, bulk soil exhibited less complexity compared to the rhizosphere (number of nodes = 343, edges = 187, average degree = 10.90). Regarding to cowpea genotypes, lines IT85F-2687 (number of nodes = 527, edges = 4607, average degree = 17.48) and IT82D-60 (number of nodes = 522, edges = 4185, average degree = 16.03) were similar. Interestingly, the cultivar BRS-Guariba showed a higher complexity (number of nodes = 420, edges = 5013, average degree = 23.87), while cultivar BRS-Tumucumaque showed the lowest complexity (number of nodes = 404, edges = 2686, average degree = 13.29). The keystone species in each network were also assessed (Supplementary Table S2). In the rhizosphere of line IT85F-2687, the top five key groups were Acidimicrobiia, *Streptomyces, Acidothermus,* Nitrososphaeraceae, and *Paenibacillus*. In line IT82D-60, the top five key species were *Bradyrhizobium,* Acidobacteria - Subgroup 6, Blastocatellaceae, Syntrophobacteraceae, and *Solirubrobacter.* Regarding cultivar BRS-Guariba, the top five key species belonged to Isosphaeraceae, *Azospirillum*, *Pirellula*, TK10, and Alicyclobacillaceae; while the key species found in the rhizosphere of cultivar BRS-Tumucumaque were Rhizobiaceae, Betaproteobacteriales, *Candidatus Udaeobacter, Conexibacter,* and Blastocatellaceae.

### Functional profile prediction of the bacterial community

The putative prediction of the potential functional profile of the soil bacterial community has shown a significant core of potential functions represented by chemoheterotrophy, aerobic chemoheterotrophy, cellulolysis, and nitrification (Supplementary Figure S2). Here, we compared the potential functions between bulk soil and the rhizosphere of each cowpea genotype, and the results revealed that these functions were significantly altered according to the genotypes. In the comparison between bulk soil and the African lines (ITF85F-2687 and IT82D-60), both increased sequences related to chemoheterotrophy. In contrast, when compared bulk soil and modern Brazilian cultivars (BRS-Guariba and BRS-Tumucumaque), bulk soil increased sequences related to cellulolysis. In general, the results have shown that African lines presented a more abundance of groups related to chemoheterotrophy, while the rhizosphere of the modern cultivars decreased potential functions related to cellulolysis.

## Discussion

**Figure 6:**
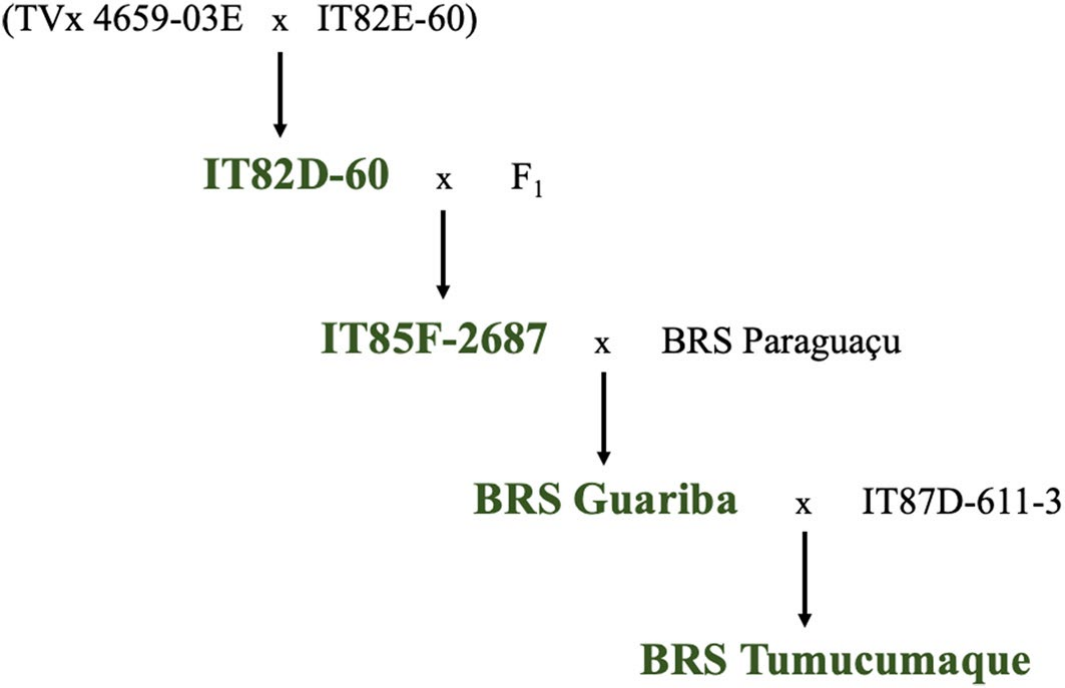
Genealogy of Brazilian cowpea cultivars (BRS-Guariba and BRS-Tumucumaque) and their African lines (IT85F-2687 and IT82D-60) parentals, used in this study.

In this study, we assessed the bacterial community of two African lines and two modern Brazilian cowpea cultivars to address the hypothesis that the breeding process affects the microbiome assembly in the rhizosphere. First, the PCA analysis showed a different bacterial community structure between bulk soil and rhizosphere. This is expected since the rhizosphere is influenced by plant exudates and promotes differences in the microbial communities’ structure when compared with the bulk soil^4,18,19^. However, the results showed that the advance of generation from African lines (IT85F-2687 and IT82D-60) to Brazilian cultivars (BRS-Guariba and BRS-Tumucumaque), accessed through their genealogies, did not affect the richness, diversity, and overall structure of the bacterial community in the rhizosphere. This similarity among the different cowpea genotypes could be explained due to their genetic similarities (Fig. 6; Table 1), as also reported by Perez-Jaramillo et al.^20^ who linked the rhizosphere microbiome composition of wild and domesticated common beans. Our results are also similar to those found by Simonin et al.^21^ who observed limited effects on the diversity and structure of microbial communities in wheat genotypes. This similarity can also be observed in the Venn diagram, which showed that 19% of the OTUs are shared between all cowpea genotypes, and these OTUs are the most abundant, accounting for 42.2% of total sequences. Although we did not find differences in the overall bacterial community structure among the different cowpea genotypes, each of them presented specific exclusive OTUs. The proportion of exclusive OTUs ranged from 7.4% in the line IT82D-60 to 18.2% in the cultivar BRS-Guaribas. Bulgarelli et al.^22^ showed that the host influence on the microbiota profile in the rhizosphere is limited, being around 5.7% of the variation attributed to the host. A similar result was found with 27 field-growth modern maize inbreeds, which showed 5-7.7% variation among cultivars^23^. Also, Mendes et al.^7^ assessed the microbial community in the rhizosphere of four common bean cultivars and found an average of 2.5% cultivar-specific OTUs. We also identified that the two modern cultivars shared more OTUs (33.6%) than the African lines (26.7%), revealing a higher similarity within the modern Brazilian cultivars.

**Table 1:** Genetic traits of cowpea genotypes (lines and cultivars).

Genotype	DM (days)	PL (cm)	SP (n^o^)	100SW (g)	DF (days)	GY (kg ha^-1^)
IT85F-2687	67	16.3	11	14.3	41	1280
IT82D-60	50	14.0	14	16.6	32	1140
BRS-Guariba	65	15.1	13	19.5	39	1480
BRS-Tumucumaque	70	22.0	15	20.0	40	1200

*DM* days for maturity, *PL* pod length, *SP* seeds per pod, *100SW* 100 seeds weight, *DF* days for flowering, *GY* grain yield.

The composition of the bacterial community showed an enrichment of the phyla Proteobacteria, Firmicutes, Bacteroidetes, and Gemmatimonadetes in the rhizosphere of cowpea. Proteobacteria consists of a group of beneficial bacteria, mainly those involved in biological N fixation, which is commonly found in leguminous plants, such as cowpea^24^. Firmicutes comprises some well-known plant growth-promoting bacteria, such as *Bacillus* and *Paenibacillus*, which are known to colonize the rhizosphere of cowpea^5^. Regarding Bacteroidetes and Gemmatimonadetes, these phyla present the ability to efficiently use carbohydrates released by the roots, being favored by root exudation^12,25^. Thus, our results showed a significant influence of rhizosphere on both bacterial phyla. Indeed, some studies with leguminous plants have found an increased abundance of these same phyla in the rhizosphere of soybean, common bean, and cowpea^5,7,20^.

In general, when considering the top 10 most abundant bacterial OTUs, which together accounted for 21.1% of the total sequences, we observed the dominance of some interesting bacterial groups in the rhizosphere. For instance, 10% of all the sequences were affiliated to the order Bacillales, which consists of a group of bacteria involved in the plant growth-promoting in the rhizosphere^5^. Also, the order Gaiellales and the genera *Ammoniphilus*, *Conexibacter*, and *Sphingomonas* are aerobic bacteria that use several organic sources that can be released by the roots^26–29^ while Xanthobacteraceae and Frankiales are recognized as N-fixers in roots of leguminous plants^30^. Thus, these bacterial groups are positively influenced by the rhizosphere environment and present a beneficial whole to the plant host.

The analysis of correlation showed that specific bacterial groups presented a negative correlation with yield. This result indicates that some bacterial groups that colonize the cowpea rhizosphere, such as *Paenibacillus*, Polyangiaceae, Nitrososphaeraceae, and Gemmataceae, do not contribute to increasing plant yield. Interestingly, *Paenibacillus* is a well-known plant growth-promoting bacteria that contributes to cowpea yield^31^, but in our study, this bacterium was negatively correlated with yield traits (seeds per pod and pod length). It may mean that this native *Paenibacillus* could differ from those improved *Paenibacillus* strains used in cowpea. Also, maybe the beneficial traits of *Paenibacillus* are not related to plant yield under the conditions of our experiment. It was previously reported that this genus induces drought tolerance and antagonizes pathogens^32^, which is not the case in this study. On the other hand, groups affiliated to Nocardioidaceae and Unclassified Chloroflexi were positively correlated with growth and productive traits. For instance, Nocardioidaceae is a family of nitrifier bacteria that acts on nitrification and contribute to increasing soil nitrate^33^. Thus, these bacteria can influence positively the growth and yield of cowpea.

We then used a co-occurrence network to disentangle the dynamics of the bacterial community in the rhizosphere of lines and cowpea cultivars. Firstly, the results indicated a less connected bacterial community in bulk soil as compared to the rhizosphere, and it agrees with previous studies that observed more connected microbial communities in the rhizosphere^34,35^. Second, there was a variation between genotypes where both African lines presented similar numbers of nodes and edges, while Brazilian cultivars were different. The bacterial community in the rhizosphere of BRS-Guariba and BRS-Tumucumaque presented the highest and lowest complexities, respectively. This result clearly shows that plant breeding affects the complexity of the rhizosphere microbiome. Rossmann et al.^14^ showed that microbial networks of wheat landraces formed a more intricate network topology than wheat cultivars, revealing a loss of microbial interactions within the rhizosphere niche. We also observed this trend in the BRS-Tumucumaque, but for the BRS-Guariba the network complexity increased. The decrease in complexity of bacterial communities in the rhizosphere of the cultivar BRS-Tumucumaque may be related to your parentage with the cultivar BRS-Guariba (one of its parentals), with loss of genetic variability (inbreeding) and due to the genetic constitution of each cultivar, which are the result of different crosses (Fig. 6). Higher network complexity is a consequence of stronger bacterial interactions in the rhizosphere^4^, and a more connected bacterial community in the rhizosphere of both lines and the cultivar BRS-Guariba indicates high interconnections between different groups of nodes, so providing higher community stability^36^. We further identified the keystone species in each network (Supplementary Table S2). Keystone groups are bacterial taxa with more betweenness centrality and are presumed to have a central role in the community^37,38^. Interestingly, most of the keystone species found in the rhizosphere of the cowpea genotypes consisted of plant growth-promoting bacteria, such as *Paenibacillus*, *Bradyrhizobium, Azospirilum,* and Rhizobiaceae. It suggests the positive effects of these keystone species on the growth and yield of cowpea. Also, some other keystone species act on N cycling or biological control in the rhizosphere, such as Nitrososphaeraceae and *Streptomyces*, respectively. Together, the results of the network analysis showed that, although the domestication process affected the dynamics of the bacterial communities, the keystone groups present a beneficial role for the plant host.

Finally, we predicted potential bacterial functions using the FAPROTAX database to verify the influence of African lines and Brazilian modern cultivars on the potential functional profile of the bacterial community. Although it is considered a potential functional prediction, this analysis has been used previously due to its suitability to understand the relationship between bacterial groups and their potential predicted functions^39–41^. Thus, the results showed ‘chemoheterotrophy’ as the dominant potential bacterial function in the rhizosphere of lines. It suggests that the bacterial groups in the rhizosphere of lines obtain energy using C sources released by the roots^42^, which was expected. Interestingly, the bacterial groups in the rhizosphere of cultivars did not show chemoheterotrophy and it could indicate that the process of plant breeding suppressed this function. In contrast, the bacterial groups from bulk soil presented the function of cellulolysis. It is interesting because cellulase is an enzyme that breaks down the cellulose into monosaccharides and as cellulose is the major constituent of plants, the function of cellulolysis can increase the degradation of plant residues in soil^43^. It is also interesting to note that the most abundant potential functions in all samples were related to the nitrogen cycle, even though they did not differ among treatments. The samples presented a high number of sequences related to nitrification, ammonia oxidation, nitrogen fixation, and nitrate reduction.

## Conclusions

In this study, we assessed the bacterial community structure and composition inhabiting the rhizosphere of different cowpea genotypes to evaluate the effect of plant breeding on the microbial community. We hypothesized plant breeding in cowpea would influence the plants’ traits and consequently drive the pattern of the bacterial community assembly in the rhizosphere. In general, our results showed a similar bacterial community in the rhizosphere of different cowpea genotypes, but with the enrichment of specific microbial groups and keystone species. We also showed that the genetic breeding process affects the dynamics of the rhizosphere community, decreasing the complexity of interaction in one cultivar. Thus, the explanation that these cowpea genotypes are genetically concordant could be applied in this study and suggest a new hypothesis of how genetic breeding of similar genotypes could influence the rhizosphere microbiome. Also, considering the importance of the microbial communities for plant growth and health, a better understanding of the microbiome assembly during the genetic breeding process will provide important information that can be useful for future plant breeding programs.

## Materials and methods

### Greenhouse experiment

The experiment was conducted in a greenhouse at the Department of Soil Science, from the Federal University of Piauí, Brazil. The climate is tropical dry with a mean precipitation of 1,300 mm yr^-1^ (with rainfall from December through May) and an annual mean temperature of 28 °C. The soil used in this study is classified as a Fluvisol, the type of soil where cowpea is commonly cultivated in Northeastern, Brazil, and it was collected at 0–20 cm depth. The soil samples were collected in an area where cowpea has been growing during the last ten years. Soil chemical properties were estimated according to Tedesco et al.^44^, being: pH – 6.1; organic C – 9.2 g kg^-1^; P – 4.9 mg kg^-1^; K – 32.5 mg kg^-1^; base saturation (V) – 58%.

 In this study, polyvinyl chloride pots (diameter 18 cm, length 16 cm) were filled with 5 kg of soil and the experiment was arranged in a completely randomized design with three replications. Two African lines (IT85F-2687 and IT82D-60) and two Brazilian cultivars (BRS-Guariba and BRS-Tumucumaque) of cowpea were selected following a generation advance and genetic breeding (Table 1; Fig. 6). Particularly, both modern cultivars are the most used by cowpea producers in Brazil^45^. All genotypes are identified and deposited in the Germplasm Bank of Cowpea from ‘Empresa Brasileira de Pesquisa Agropecuária – EMBRAPA’, in Teresina (Embrapa Meio-Norte), Piauí, Brazil. The permission to use these genotypes was obtained from Embrapa Meio-Norte. Five seeds of each genotype (lines and modern cultivars) were sown per pot and ten days after germination, plants were thinned, leaving one individual plant per pot. Pots were irrigated daily with sterilized water to maintain soil moisture at 80% of field capacity. The temperature of the greenhouse was controlled at 30 °C.

### Rhizosphere sampling

Plants were collected 40 days after emergence (corresponding to the flowering period). Rhizospheric soil sampling was performed as follows: roots and adhering soil of each plant were placed onto a 1 mm mesh sieve and washed thoroughly with a gentle stream of tap water to remove the soil. All soil samples were stored at − 20 °C prior to DNA extraction and analysis.

### DNA extraction and sequencing

DNA was extracted from 0.5 g (total humid weight) of soil using the Power Lyzer Power Soil DNA Isolation Kit (MoBIO Laboratories, Carlsbad, CA, USA), according to the manufacturer’s instructions. The DNA extraction was performed in triplicate for each soil sample. The quality and concentration of the extracted DNA were determined by using NanoDrop 2000 spectrophotometer (Thermo Scientific, Waltham, USA).

The V4 region of the 16S rRNA gene was amplified with region-specific primers (515F/806R)^46^. Each 25 μl PCR reaction contained the following: 12.25 μL of nuclease-free water (Certified Nuclease-free, Promega, Madison, WI, USA), 5.0 μL of buffer solution 5x (MgCl_2_ 2Mm), 0.75 μL of solution of dNTP’s (10 mM), 0.75 μL of each *primer* (515 YF 40 μM e 806 R 10μM), 1.0 unit of Platinum Taq polymerase High Fidelity in a concentration of 0.5 μL (Invitrogen, Carlsbad, CA, USA), and 2.0 μL of template DNA. Moreover, a control reaction was performed by adding water instead of DNA. The conditions for PCR were as follows: 95 °C for 3 min to denature the DNA, with 35 cycles at 98°C for 20 s, 55 °C for 20 s, and 72 °C for 30 s, with a final extension of 3 min at 72°C to ensure complete elongation.

After indexing, the PCR products were cleaned up using Agencourt AMPure XP – PCR purification beads (Beckman Coulter, Brea, CA, USA), according to the manufacturer’s manual, and quantified using the dsDNA BR assay Kit (Invitrogen, Carlsbad, CA, USA) on a Qubit 2.0 fluorometer (Invitrogen, Carlsbad, CA, USA). Once quantified, equimolar concentrations of each library were pooled into a single tube. After quantification, the molarity of the pool was determined and diluted to 2 nM, denatured, and then diluted to a final concentration of 8.0 pM with a 20% PhiX (Illumina, San Diego, CA, USA) spike for loading into the Illumina MiSeq sequencing machine (Illumina, San Diego, CA, USA).

Sequence data were processed using QIIME 2 version 2019.10. Firstly, the sequences were demultiplexed and quality control was carried out using DADA2^47^, using the consensus method to remove any remaining chimeric and low-quality sequences. After filtering, approximately 355,000 high-quality sequences were obtained, with an average of ~19,600 sequences per sample. Singletons and doubletons were removed, and the samples were rarefied to 8600 sequences, following the number of the lowest sample. The taxonomic affiliation was performed at 97% similarity using the Silva database v. 132^48^, and the generated matrix was further used for statistical analyses. The sequences were submitted to the NCBI Sequence Read Archive under the identification PRJNA751574.

### Data analysis

To compare the bacterial community structure between treatments we used Principal component analysis. First, the data matrix was initially analyzed using Detrended correspondence analysis (DCA), indicating a linear data distribution and the best-fit mathematical model was the PCA. To test whether the different niches (i.e., bulk soil and rhizosphere) and cowpea genotypes harbored significantly different active bacterial communities we conducted a two-way permutational multivariate analysis of variance (PERMANOVA). PCA analysis was conducted using Canoco 4.5 (Biometrics, Wageningen, The Netherlands) and PERMANOVA using Past v.4^49^. We also used Past to calculate the richness of observed OTUs and Shannon’s diversity index, and the comparison was based on Tukey’s HSD test. A Venn diagram was constructed to verify the proportion of groups exclusive and shared between treatments using the webtool Venny 2.1^50^. To determine the differential composition of bacterial groups among treatments, we used the software STAMP^51^. P-values were calculated based on two-sided Welch’s t-test, and correction using Benjamini-Hochberg FDR. In addition, to investigate the correlation between bacterial groups and cowpea plant performance properties we used Spearman’s rank correlation coefficient using R^52^. We then used network analysis to assess the complexity of interactions among bacterial taxa in each treatment. For this, non-random co-occurrence analysis was carried out using the Python module ‘SparCC’^53^. For this, a table of frequency of OTUs was used for analysis, and SparCC correlations were calculated, and only strong (>0.9 or <− 0.9) and highly significant (*p* < 0.01) correlations were selected. The nodes in the reconstructed network represent OTUs, whereas the edges represent significantly positive or negative correlations between nodes. The network graphs were based on a set of measurements, including the number of nodes, number of edges, modularity, number of communities, average node connectivity, average path length, diameter, and cumulative degree distribution. The network visualization and properties measurements were calculated with the interactive platform Gephi^54^. Finally, to further predict the relevant potential functions performed by the bacterial community, we performed a functional annotation using FAPROTAX^53^, a database that maps prokaryotic clades (e.g. genera or species) to established metabolic or other ecologically relevant functions, based on cultured strains. For this, a table of frequency of taxa at the genus level was used as input and converted into a putative functional table and the comparisons were done between bulk soil and the rhizosphere of the different cowpea genotypes.

## Supplementary Information

Supplementary Information 1.

Supplementary Information 2.
