# Supplementary material for: Genetically related genotypes of cowpea present similar bacterial community in the rhizosphere
Source: Sci Rep. 2022 Mar 2;12:3472. doi: 10.1038/s41598-022-06860-x (PMC8891268; doi:10.1038/s41598-022-06860-x)
Supplement: Supplementary file 1 — Supplementary Information 1. [file 41598_2022_6860_MOESM1_ESM.docx]

**Genetically related genotypes of cowpea present similar bacterial community in the rhizosphere**

Tayna Mendes de Albuquerque, Lucas William Mendes, Sandra Mara Barbosa Rocha, Jadson Emanuel Lopes Antunes, Louise Melo de Souza Oliveira, Vania Maria Maciel Melo, Francisca Andrea Silva Oliveira, Arthur Prudêncio de Araujo Pereira, Veronica Brito da Silva, Regina Lucia Ferreira Gomes, Francisco de Alcantara Neto, Angela Celis de Almeida Lopes, Maurisrael de Moura Rocha, Ademir Sérgio Ferreira Araujo


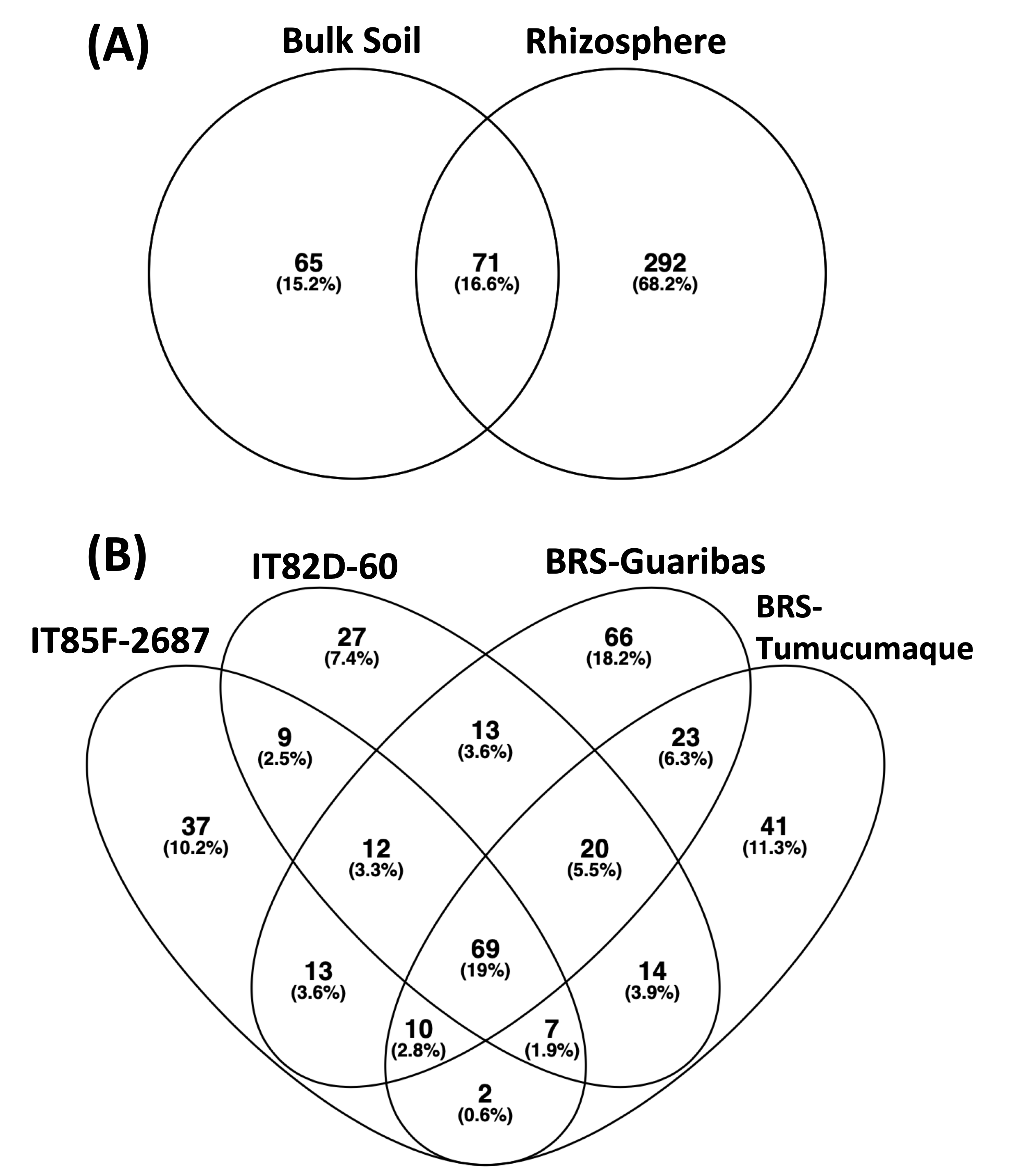


**Supplementary Figure S1.** Venn diagrams showing the exclusive and shared OTUs (A) between bulk soil and cowpea rhizosphere and (B) between the rhizosphere of different cowpea genotypes.


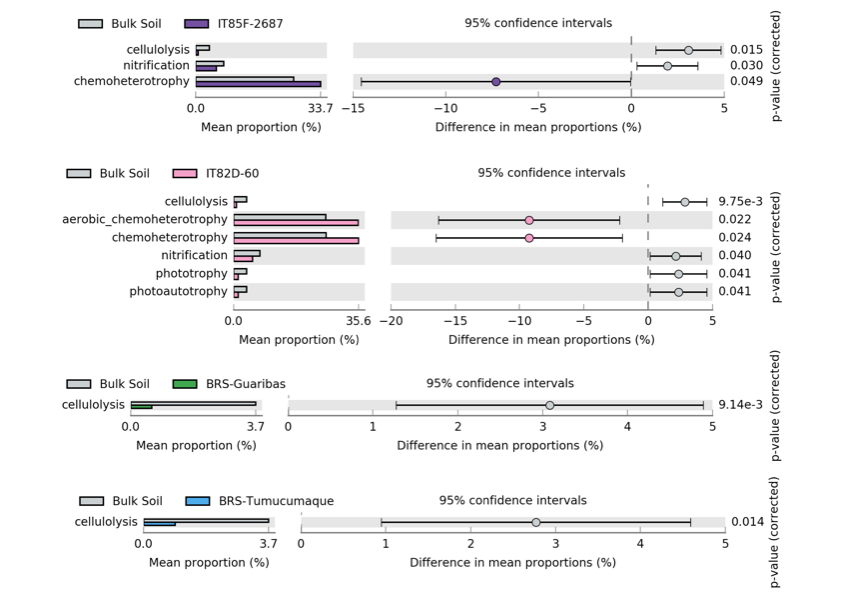


**Supplementary Figure S2.** Scatter-plots showing the differential abundance of putative functions between bulk soil and the different cowpea genotypes. The significance is based on Welch's t-test with Benjamini-Hochberg correction (*p* < 0.05). The classification in the figure is based on the functional prediction on the FAPROTAX database.
