# Supplementary material for: Genetically related genotypes of cowpea present similar bacterial community in the rhizosphere
Source: Sci Rep. 2022 Mar 2;12:3472. doi: 10.1038/s41598-022-06860-x (PMC8891268; doi:10.1038/s41598-022-06860-x)
Supplement: Supplementary file 2 — Supplementary Information 2. [file 41598_2022_6860_MOESM2_ESM.docx]

**Genetically related genotypes of cowpea present similar bacterial community in the rhizosphere**

Tayna Mendes de Albuquerque, Lucas William Mendes, Sandra Mara Barbosa Rocha, Jadson Emanuel Lopes Antunes, Louise Melo de Souza Oliveira, Vania Maria Maciel Melo, Francisca Andrea Silva Oliveira, Arthur Prudêncio de Araujo Pereira, Veronica Brito da Silva, Regina Lucia Ferreira Gomes, Francisco de Alcantara Neto, Angela Celis de Almeida Lopes, Maurisrael de Moura Rocha, Ademir Sérgio Ferreira Araujo

**Supplementary Table 1.** Correlations and topological properties of microbial networks in bulk soil and rhizosphere of four cowpea genotypes.

| **Network property** | **Bulk** | **IT85F-2687** | **IT82D-60** | **BRS-Guariba** | **BRS-Tumucumaque** |
| --- | --- | --- | --- | --- | --- |
| Number of nodes^a^ | 343 | 527 | 522 | 420 | 404 |
| Number of edges^b^ | 1870 | 4607 | 4185 | 5013 | 2686 |
| Positive edges^c^ | 1595 (85%) | 2777 (60%) | 2808 (67%) | 3258 (65%) | 2161 (80%) |
| Negative edges^d^ | 275 (15%) | 1830 (40%) | 1377 (33%) | 1755 (35%) | 525 (20%) |
| Modularity^e^ | 0.933 | 2.414 | 1.638 | 1.663 | 1.013 |
| Number of communities^f^ | 26 | 20 | 52 | 31 | 31 |
| Network diameter^g^ | 22 | 17 | 14 | 12 | 11 |
| Average path length^h^ | 7.126 | 6.463 | 5.933 | 4.868 | 5.020 |
| Average degree^i^ | 10.90 | 17.48 | 16.03 | 23.87 | 13.29 |
| Av. clustering coefficient^j^ | 0.343 | 0.300 | 0.294 | 0.434 | 0.378 |

^a^Microbial taxon (at genus level) with at least one significant (*p* < 0.01) and strong (SparCC > 0.7 or < -0.7) correlation;

^b^Number of connections/correlations obtained by SparCC analysis;

^c^SparCC positive correlation (> 0.7 with *p* < 0.01);

^d^sparcc negative correlation (< -0.7 with *p* < 0.01);

^e^The capability of the nodes to form highly connected communities, that is, a structure with high density of between nodes connections (inferred by Gephi);

^f^A community is defined as a group of nodes densely connected internally (Gephi);

^g^The longest distance between nodes in the network, measured in number of edges (Gephi);

^h^Average network distance between all pair of nodes or the average length off all edges in the network (Gephi);

^i^The average number of connections per node in the network, that is, the node connectivity (Gephi);

^j^How nodes are embedded in their neighborhood and the degree to which they tend to cluster together (Gephi);

Meth-Meth: correlation within methanotrophs. Meth-Non: correlation between methanotrophs and non-methanotrophs. Non-Non: correlation between non-methanotrphs.

**Supplementary Table 2.** Top 10 OTUs with more betweenness centrality and number of correlations (that is, degree) in bulk soil and rhizosphere of four cowpea cultivars.

|  | **OTU** | **Classification (lowest level)** | **Beteweeness Centrality** | **Degree** |
| --- | --- | --- | --- | --- |
| **Bulk soil** | OTU_9 | Actinobacteria - *Conexibacter* | 27451.19 | 15 |
|  | OTU_214 | Planctomycetes - Isosphaeraceae | 18169.61 | 5 |
|  | OTU_380 | Planctomycetes - Gemmataceae | 15451.47 | 15 |
|  | OTU_762 | Acidobacteria - Blastocatellia (Subgroup 4) | 14430.01 | 13 |
|  | OTU_609 | Planctomycetes - Tepidisphaerales | 12158.40 | 12 |
|  | OTU_1084 | Planctomycetes - Gemmataceae | 10311.78 | 7 |
|  | OTU_485 | Acidobacteria - *Acidithiobacillus* sp. | 9900.73 | 5 |
|  | OTU_1 | Firmicutes - Bacillales | 9900.02 | 12 |
|  | OTU_755 | Unclassified Bacteria | 9172.36 | 30 |
|  | OTU_72 | Proteobacteria - Rhizobiales | 6919.83 | 15 |
| **IT85F-2687** | OTU_359 | Actinobacteria - Acidimicrobiia | 67959.21 | 13 |
|  | OTU_27 | Actinobacteria - *Streptomyces* | 57946.11 | 4 |
|  | OTU_268 | Actinobacteria - *Acidothermus* | 43023.55 | 9 |
|  | OTU_117 | Thaumarchaeota - Nitrososphaeraceae | 35798.25 | 12 |
|  | OTU_286 | Firmicutes - *Paenibacillus* | 31883.46 | 26 |
|  | OTU_222 | Gemmatimonadetes - Gemmatimonadaceae | 31632.71 | 4 |
|  | OTU_433 | Chloroflexi - Roseiflexaceae | 30243.00 | 11 |
|  | OTU_46 | Proteobacteria - Rhizobiaceae | 26499.57 | 8 |
|  | OTU_100 | Acidobacteria - Blastocatellaceae | 21071.98 | 84 |
|  | OTU_31 | Actinobacteria - Gaiellales | 19265.39 | 35 |
| **IT82D-60** | OTU_24 | Proteobacteria - *Bradyrhizobium* | 55434.54 | 9 |
|  | OTU_22 | Acidobacteria - Subgroup 6 | 54754.75 | 9 |
|  | OTU_128 | Acidobacteria - Blastocatellaceae | 36927.44 | 13 |
|  | OTU_37 | Proteobacteria - Syntrophobacteraceae | 31024.50 | 10 |
|  | OTU_14 | Actinobacteria - *Solirubrobacter* | 28014.26 | 38 |
|  | OTU_232 | Acidobacteria - Blastocatellaceae | 25876.21 | 21 |
|  | OTU_234 | Proteobacteria - Pseudomonadales | 12305.52 | 15 |
|  | OTU_3 | Firmicutes - *Bacillus* | 11406.72 | 52 |
|  | OTU_63 | Proteobacteria - *Dyella* | 9798.72 | 24 |
|  | OTU_32 | Proteobacteria - Myxococcales | 8436.77 | 20 |
| **BRS-Guaribas** | OTU_225 | Planctomycetes - Isosphaeraceae | 15649.71 | 11 |
|  | OTU_575 | Proteobacteria - Azospirillum | 15311.19 | 21 |
|  | OTU_1311 | Planctomycetes - Pirellula | 13263.22 | 8 |
|  | OTU_26 | Chloroflexi - TK10 | 12511.55 | 22 |
|  | OTU_8 | Firmicutes - Alicyclobacillaceae | 10355.19 | 22 |
|  | OTU_196 | Nitrospirae - *Nitrospira* | 9972.06 | 41 |
|  | OTU_1336 | Proteobacteria - Gammaproteobacteria | 9542.84 | 2 |
|  | OTU_106 | Actinobacteria - Gaiellales | 8338.62 | 22 |
|  | OTU_1106 | Planctomycetes - *Pirellula* | 7709.26 | 15 |
|  | OTU_28 | Proteobacteria - *Burkholderia* | 7292.31 | 11 |
| **BRS-Tumucumanaque** | OTU_46 | Proteobacteria - Rhizobiaceae | 11590.93 | 9 |
|  | OTU_25 | Proteobacteria - Betaproteobacteriales | 10520.99 | 20 |
|  | OTU_18 | Verrucomicrobia - *Candidatus Udaeobacter* | 9985.13 | 7 |
|  | OTU_9 | Actinobacteria; *Conexibacter* | 9540.66 | 11 |
|  | OTU_17 | Acidobacteria - Blastocatellaceae | 8819.84 | 18 |
|  | OTU_281 | Acidobacteria - Subgroup 6 | 8780.02 | 17 |
|  | OTU_303 | Proteobacteria - *Azospirillum* | 8761.01 | 27 |
|  | OTU_3 | Firmicutes - *Bacillus* | 7955.09 | 18 |
|  | OTU_48 | Actinobacteria - Gaiellales | 7608.61 | 30 |
|  | OTU_402 | Chloroflexi | 5519.50 | 8 |
